# Trend Prediction and Operation Alarm Model Based on PCA-Based MTL and AM for the Operating Parameters of a Water Pumping Station

**DOI:** 10.3390/s24165416

**Published:** 2024-08-21

**Authors:** Zhiyu Shao, Xin Mei, Tianyuan Liu, Jingwei Li, Hongru Tang

**Affiliations:** School of Electrical, Energy and Power Engineering, Yangzhou University, No. 88 South University Road, Yangzhou 225009, China; mxin0103@163.com (X.M.); ltianyuan2022@163.com (T.L.); jingwei_li@yzu.edu.cn (J.L.); yztanghr@163.com (H.T.)

**Keywords:** pumping station unit monitoring, predictive models, PCA-based multi-task learning, attention mechanism

## Abstract

In order to effectively predict the changing trend of operating parameters in the pump unit and carry out fault diagnosis and alarm processes, a trend prediction model is proposed in this paper based on PCA-based multi-task learning (MTL) and an attention mechanism (AM). The multi-task learning method based on PCA was used to process the operating data of the pump unit to make full use of the historical data to extract the key common features reflecting the operating state of the pump unit. The attention mechanism (AM) is introduced to dynamically allocate the weight coefficient of common feature mapping for highlighting the key common features and improving the prediction accuracy of the model when predicting the trend of data change for new working conditions. The model is tested with the actual operating data of a pumping station unit, and the calculation results of different models are compared and analyzed. The results show that the introduction of multi-task learning and attention mechanisms can improve the stability and accuracy of the trend prediction model compared with traditional single-task learning and static common feature mapping weights. According to the threshold analysis of the monitoring statistical parameters of the model, a multi-stage alarm model of pump unit operation condition monitoring can be established, which provides a theoretical basis for optimizing operation and maintenance management strategy in the process of pump station management.

## 1. Introduction

The pumping station is an important facility within water conservancy projects, providing essential water resources for domestic use, agricultural irrigation, and industrial production. The safe operation of pumping stations is crucial for the consistent functioning of water conservancy projects and the protection of lives and property. However, during the operation of pumping station systems, factors such as equipment aging, environmental changes, and improper operation can result in equipment failures, system performance degradation, and ultimately accidents such as resource waste, equipment damage, and personnel injury. Therefore, it is necessary to monitor the real-time operational status of pumping stations and diagnose and alert any irregularities in the operation of pumping station units. This approach ensures the continuous, safe, and stable operation of pump unit equipment within pumping stations, reduces equipment failure rates, enhances maintenance and inspection efficiency, and optimizes management strategies. To achieve these goals, the most widely used and effective approach is to develop models that predict the trends of pumping station unit parameters and alert management when units may operate abnormally.

The trend prediction of pumping station unit operating parameters is based on analyzing various real-time operational data of these units. This process involves the real-time analysis and trend prediction of key parameters that may affect the health and safe operation of pumping station units, identifying potential fault hazards in advance for equipment diagnosis and maintenance management [1,2]. Researchers have extensively studied monitoring the status of pumping station unit equipment and predicting the trends of operating parameters based on data analysis.

In terms of monitoring the status of pump station equipment, there are three popular methods for monitoring the status of water pump stations: model-based methods, signal analysis-based methods, and data-driven methods [3]. Model-based methods establish accurate physical models based on the operating mechanism of unit equipment to reflect the operating status of the unit, which usually ensures high accuracy [4]. Data from the pumping station’s operation processes (such as electrical parameters, pressure or flow in pipelines, water levels in tanks, and changes in discrete states) represent a valuable resource for operation management. Marko highlighted the importance and advantages of employing hybrid models with suitable “data-driven” techniques for controlling water supply systems [5]. With the development of digital twin technology, many researchers have begun to construct digital twin models for pumping stations. Hallaji proposed a digital twin framework to extend the scope of predictive maintenance by leveraging building information modeling and deep learning [6]. Feng developed a high-precision digital twin modeling method tailored for pumping stations, which could complete the automatic inspection of the pumping station; optimization of scheduling, prediction and regulation of energy, and carbon emissions; and visualization of results for display and other applications [7]. However, the model-based method also has its drawbacks. For the operation of units in complex scenarios, such methods make it difficult to establish accurate physical models [8]. In addition, due to the wear and tear during use, the physical operating mechanism of the equipment may change, resulting in changes in the operating model parameters. Operating models that cannot be updated synchronously may lead to false alarms or missed alarms. Therefore, some researchers use signal analysis-based methods for monitoring. Fu investigated a novel hybrid approach combining a multiscale dominant ingredient chaotic analysis, kernel extreme learning machine, and adaptive mutation grey wolf optimizer for predicting vibration trends in hydropower generator units [9]. In Wang’s work for the real-time analysis and processing of data in pumping station operation and maintenance systems, a hybrid prediction method was proposed to predict the vibration responses of the pumping station based on a single model of the autoregressive integrated moving average (ARIMA), a combined model of the adaptive network-based fuzzy inference system (ANFIS) and whale optimization algorithm (WOA) [10]. Signal analysis-based methods detect potential faults by processing and analyzing collected signal data, making them sensitive to signal quality and highly susceptible to noise and interference. Thus, specialized sensors and devices are required, which will increase operating costs. Moreover, some key signals are difficult or impossible to collect. Thus, the application range of signal analysis-based methods is limited [11]. Consequently, more researchers prefer to use data-driven methods to monitor pumping station devices. Data-driven methods do not rely on physical models and can adapt to the differences of various systems and devices. The migration and deployment of models are convenient, reducing development and maintenance costs. Data-driven methods include methods based on statistical analysis [12] and those based on machine learning [13]. Statistical analysis-based methods rely on data distribution characteristics, monitoring the operation status through calculated characteristic parameters based on establishing the relationship between variables. This method requires researchers to have rich experience and professional knowledge of pumping stations when judging the relationships between variables and screening useful features. Machine learning-based methods typically require the development of complex network structures and the tuning of numerous hyperparameters, making it challenging to explain and understand the model’s decision-making process. In addition, such methods require certain hardware facilities for computation. More importantly, when the number of training samples is not sufficient, such methods are prone to overfitting, and their generalization ability is difficult to guarantee.

In the research of data analysis and trend prediction algorithms, methods and models from other fields have been extensively applied in water conservancy engineering. The main goal of analyzing the operating parameter data of pumping station equipment is to extract meaningful insights from real-time or historical data, explain and discover potential patterns or correlations between variables, obtain a deeper understanding of system operating characteristics, and predict the trend of parameter operation. Previous studies have mainly used traditional statistical methods, such as calculating the mean and analysis of variance, to analyze the operating data of pumping stations, but these methods have encountered difficulties in dealing with complex and time-varying pumping station systems. In recent years, machine learning (ML) methods have become an important tool for pump station data analysis. The application of methods such as support vector machines, random forests, and neural networks improves the accuracy of data analysis by effectively handling nonlinear relationships and complex correlations between multiple variables. For example, Surucu reviewed the recent literature on ML-driven condition monitoring systems that have been beneficial in many cases and provided insights into the underlying findings on successful, intelligent condition monitoring systems [14]. Eiben compared the effectiveness of random forest and k-means clustering models in predicting failures at pumping stations [15]. Khorsheed explored the integration of machine learning with decision-making techniques to predict potential bearing failures, thereby improving overall manufacturing operations by enabling timely maintenance actions [16]. At the same time, time series analysis methods such as ARIMA [17] and exponential smoothing [18] have been widely adopted to capture trends and seasonal changes in the time series data of pumping stations in order to achieve long-term operational prediction and analysis.

At present, there are several prominent trends in the development of analysis and trend prediction for pump station data. Firstly, the application of deep learning technology will be further expanded, and models such as deep neural networks [19], long short-term memory networks (LSTMs) [20], and convolutional neural networks (CNNs) [21,22] will improve their modeling and prediction capabilities for complex systems. Secondly, the integration of edge computing and Internet of Things technology will become an important direction [23,24]. By enabling data processing at the device level, more efficient data management and analysis can be achieved. In addition, future research should focus more on the fusion and integration of multi-source data to establish a more comprehensive pump station information model. With the development of artificial intelligence technology, pump station data analysis will further move towards intelligent decision-making systems, achieving the automated operation and intelligent optimization of pump station systems.

The operational status of pumping stations is represented by multiple variables, necessitating the use of multivariate statistical analysis methods in the data analysis and modeling process. It is usually necessary to perform dimensionality reduction on the data to extract key features to monitor and judge the performance of the water pump unit. Principal component analysis (PCA) is the most commonly used method that uses a large amount of data to ensure its statistical characteristics and accurately captures the main direction of data changes [25]. The pump unit in a pumping station operates intermittently and changes gradually, with each operating period being considered as a separate task. As the operating years increase, the number of tasks grows, but the low frequency of data collection results in limited effective data for analysis within each task, presenting challenges for data analysis. Therefore, how to monitor the status of water pump units under the condition of multiple tasks and few samples has become a significant research focus. The machine paradigm of multi-task learning (MTL) improves the model performance by simultaneously learning multiple related tasks, offering a solution to the problem of multiple tasks with few samples [26,27]. In traditional single-task learning, a model is trained to solve a specific task. In multi-task learning, models are designed to handle multiple tasks simultaneously, which can relate and share certain features to improve the generalization performance of the model. Achille et al. [28] addressed the challenge of task description in multi-task learning, while Zhang et al. [29] explored the use of geometric reasoning, scene depth, and semantics to optimize the effectiveness of multi-task learning. In the analysis and prediction of operating parameters for pumping station equipment, correlations between different monitoring tasks—such as vibration and electrical parameters—can be analyzed to extract appropriate features that represent each task. Moreover, the characteristics of monitoring data for pump stations operating under different conditions can vary significantly. Therefore, uncertainty information can be considered to adjust the weight of model parameters to adapt to different situations, aiming to improve the generalization ability of the model and enable the model to adapt to monitoring tasks under different operating conditions, pump station unit types, or environments. In the process of multi-task learning, each task utilizes all of the extracted features. However, due to factors such as seasonal changes and noise interference, features may shift. Moreover, not every feature will exist in each task; in other words, some tasks may only have specific features. Therefore, how to select the features extracted by multi-task learning has become another problem that needs to be solved. Introducing an attention mechanism (AM) in the process of multi-tasking learning is a feasible method. The attention mechanism simulates the mechanism of human attention allocation in information processing, allowing the model to allocate different weights based on different parts of the input that are focused when processing data, thereby more flexibly dealing with complex tasks and data [30]. The attention mechanism has been widely applied in fields such as image processing and speech recognition, but it is less commonly used in the field of pump station data analysis and processing. In pump station operation monitoring, different sensors and monitoring tasks can provide information about different aspects of the pump station system. The model makes it difficult to effectively focus on the key information in the large input data. Therefore, attention mechanisms need to be introduced to help the models dynamically pay attention to the data of different monitoring tasks and adjust the weights of tasks according to the current situation. This will be beneficial for improving the adaptability of the monitoring system to multiple tasks, ensuring a more comprehensive understanding of the operating status of the water pump system.

Thus, based on the above background, a trend prediction model based on PCA-based MTL and AM for the operating parameters of water pumping stations is proposed in this paper. The multi-task learning method based on PCA was used to process the operating data of the water pumping station to make full use of the historical data to extract the key common features reflecting the operating state of the devices. The attention mechanism is introduced to dynamically allocate the weight coefficient of common feature mapping for highlighting the key common features and improving the prediction accuracy of the model when predicting the trend of data change for new working conditions.

## 2. Methods

The basic process of the trend prediction model based on the PCA-based multi-task learning and attention mechanism for operating parameters of the water pumping station in this article is as follows: Firstly, the multi-task learning method based on PCA is used to reduce the dimensionality of the monitoring data and filter out common features of the historical operating condition and tasks. Then, the attention mechanism is introduced to dynamically determine and adjust the weights of the prediction model in each common feature direction. Finally, the real-time operating condition data are mapped to the principal component direction of the common features, and the trend of changes is predicted based on the characteristics and variations in the principal component direction. Two statistical parameters are used to evaluate the predictive performance of the model in the process of training and testing, and they are also used as thresholds to determine whether the operating state of water pumping units is abnormal and what level of alert is issued when the status is abnormal.

### 2.1. PCA-Based Multi-Task Learning

The operation of the water pumping unit is not continuous, and each operating interval can be treated as a separate task. Therefore, large amounts of historical data can be fully utilized to enable the model to learn the characteristics of data changes during pump station operation. Multi-task learning can improve the performance of the predictive model by simultaneously learning multiple related tasks. At the same time, there are many types of variables in the operation data of pumping stations, and long-term operation and slow parameter changes cause data redundancy, so dimensionality reduction processing is needed. The historical operating data of the pumping units can be represented as $X=\left\{X_{1},X_{2},\cdots X_{k}\cdots ,X_{N}\right\}$, where *N* represents the number of groups of data, each group of data $X_{k}$ is a matrix of size $N_{k}\times m$, $N_{k}$ is the sample size of the group, and *m* is the number of features monitored of the device. The process of standardization is necessary to ensure that the results of the PCA accurately capture the main direction of data changes and are not affected by different feature scales. The covariance matrix C in the PCA can represent the correlation between different features, thereby identifying the main direction of change in the data. It reflects the degree of linear relationship between the various features of the data. The covariance matrix C can be expressed as $C=U\Sigma V^{T}$ through singular value decomposition, so X can be written as the sum of the outer products of *k* vectors:

(1) $$X=t_{1}p_{1}^{T}+t_{2}p_{2}^{T}+\ldots +t_{k}p_{k}^{T}=TP^{T}$$

where T=UΣ = $\left[t_{1},t_{2},\ldots ,t_{k}\right]$ is called the score matrix and P=V = $\left[p_{1},p_{2},\ldots ,p_{k}\right]$ is called the load matrix. By performing the above operation on *N* sets of historical operating data, we can obtain the set of principal component numbers $K_{k}=\left\{k_{1},k_{2},\ldots ,k_{N}\right\}$, the set of score matrix $T_{k}=\left\{T_{1},T_{2},\ldots ,T_{N}\right\}$, and the set of load matrix $P_{k}=\left\{P_{1},P_{2},\ldots ,P_{N}\right\}$. In PCA, if two vectors have the same direction, it means that they have similar trends in the principal component direction of the data, that is, their directions of change with the data are similar. The cosine similarity can be used to measure the degree of similarity in the direction between two vectors, so the degree of similarity in the direction between each principal component vector in the set of load matrix $P_{k}$ can be calculated as:

(2) $$\cos\left(\alpha_{\mathrm{di}},\alpha_{\mathrm{tj}}\right)=\frac{\alpha_{\mathrm{di}}\cdot \alpha_{\mathrm{tj}}}{\|\alpha_{\mathrm{di}}\left\|\cdot \right\|\alpha_{\mathrm{tj}}\|},d,t\in \left\{1,\ldots ,N\right\}$$

where $\alpha_{di}$ is the ***i***-th principal component in the d-th load matrix. $\alpha_{tj}$ is the ***j***-th principal component in the ***t***-th load matrix. The closer the cosine similarity is to 1, the closer these two principal components are to each other. If $\left|\cos\left(\alpha_{di},\alpha_{tj}\right)\right|\geq \varsigma$, where ς indicates the proportion of total variance that we wish to retain (usually taken as 90% or 95%), it means that their trends in the main direction of data change are sufficiently similar and contain similar information. Thus, we can obtain a set of columns with consistent directions in *q* groups, and there are $n_{q}$ vectors with consistent directions in each group $\left\{\alpha_{1},\ldots ,\alpha_{n_{q}}\right\}$. By splicing it and conducting SVD decomposition: $A=\left[\alpha_{1},\alpha_{2},\cdot \cdot \cdot ,\alpha_{n_{q}}\right]$, $AA^{T}=U\Sigma V^{T}$. If the first column of orthogonal matrix U is treated as a new direction $\beta_{1}$, the $\beta_{1}$ represents the direction corresponding to the maximum singular value of $AA^{T}$, which is the main direction of change in the data. In this way, *q* new directions can be obtained, which can be used as a comprehensive feature to represent the main changing directions of the original data. $W=\left[\beta_{1},\;\beta_{2},\cdot \cdot \cdot ,\beta_{q}\right]$ can serve as common features of these *N* sets of historical operating data of the water pumping unit, which are the common features obtained in PCA-based multi-task learning and more comprehensively reflect the changing characteristics of the data.

In summary, the PCA algorithm based on multi-task learning is shown as follows:

Step 1: Define and standardize *N* sets of historical operating conditions data for the equipment;
Step 2: Calculate the covariance matrix C;
Step 3: Calculate the score matrix T;
Step 4: Calculate the load matrix P;
Step 5: Calculate the cosine similarity values between the column vectors in P to obtain the vectors with $\left|\cos\right|\geq \varsigma$ and calculate W.

### 2.2. Weight Adjustment Based on the Attention Mechanism

**Figure 1:**
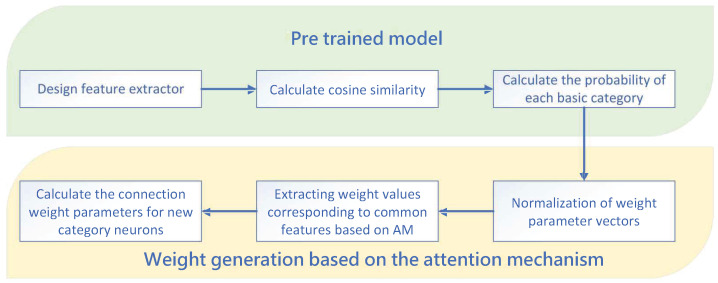
Process of weight generation based on the attention mechanism. The weight generator consists of two parts: the pre-trained model and the weight generation based on the attention mechanism.

After obtaining the common features from the PCA-based multi-task learning, the next step is to map the data of the new operating period of the device onto these common features to further analyze their changing trends in the common directions. Different sensors and monitoring tasks can provide information about different aspects of the pump station system. The model makes it difficult to effectively focus on the effective key information in the large input data. Thus, introducing attention mechanisms is essential to enable the model to dynamically focus on the data from different monitoring tasks [31]. The process of weight generation based on the attention mechanism is shown in Figure 1. It comprises two parts: (1) The pre-trained model. This part uses convolutional neural networks to analyze historical data and classify existing categories. (2) The weight generation based on the attention mechanism. This section promotes the commonalities obtained through training tasks and provides weight parameter values for new tasks.

The pre-trained model consists of three steps:

Step 1: Design the feature extractor:

(3) z=F(X|θ)

where *X* is the training dataset of the tasks, **z** is the feature set of the dataset *X* output by the feature extractor, and θ are trainable parameters in the feature extractor.

Step 2: Calculate the cosine similarity:

(4) $$s=\tau \frac{z}{\left\|z\right\|}\cdot \frac{W^{*}}{\|W^{*}\|}$$

where τ is a trainable constant parameter, $W^{*}$ represents the $L_{2}$ norm of weight values of the last layer in the pre-trained model, where each element represents the weight parameters of $K^{*}$ basic categories. The basic categories refer to the categories included in the trained tasks.

Step 3: Calculate the cosine similarity: Calculate the probability p of each basic category:

(5) $$p=Soft\max\left(s\right)$$

where each element of p represents the probability that *X* belongs to $K^{*}$ basic categories, respectively.

The weight generator based on the attention mechanism also includes three steps:

Step 1: Design the feature extractor: In the pre-trained model, $W^{*}$ is the weight parameter vector, which refers to the weight parameters of all connections of the last layer of neurons. After normalizing $W^{*}$ using the $L_{2}$ norm of the last layer’s weight values, these weight values $W_{1}^{*},W_{2}^{*},\cdot \cdot \cdot ,W_{K}^{*}$ are stored in the memory module, where the number of neurons in the last layer is *K*; each element in the feature set $k_{1},k_{2},\cdot \cdot \cdot ,k_{K}$ of the trained task dataset represents the corresponding feature of the category dataset.

Step 2: In the memory module, use the attention mechanism to extract the weight values corresponding to the most relevant features and perform weighted averaging:

(6) $${W^{\prime }}_{att}=\frac{1}{m}\sum_{i=1}^{m}\sum_{b=1}^{K}A\left(\phi_{q}\frac{{z^{\prime }}_{i}}{\|{z^{\prime }}_{i}\|},k_{b}\right)\cdot \frac{{W_{b}}^{*}}{\|{W_{b}}^{*}\|}$$

where *m* is the number of sample points in the new task training set, *K* is the number of categories in the trained tasks, and $\phi_{q}$ is the trainable parameter. ${W_{b}}^{*}$ are all the connection weight parameters corresponding to the *b*-th class neuron in the last layer of the pre-trained deep network. $z_{i}^{\prime }$ is the feature of the dataset for the *i*-th sample point in the new task. $k_{b}$ is the feature of the dataset of the *b*-th category in the memory module. ${W^{\prime }}_{att}$ is the weighted average of the weight parameters corresponding to the most relevant features extracted from the memory module by the attention mechanism. A represents the attention mechanism, which is the similarity among the features of each sample task in the new task and the features of each category in the memory module. The greater the similarity between them, the greater the value of A, which indicates that the connection weight parameters of neurons of this category have a greater weight. The value of A can be calculated using the following formula:

(7) $$A\left(\phi_{q}\frac{{z^{\prime }}_{i}}{\|{z^{\prime }}_{i}\|},k_{b}\right)=Soft\max\left(\phi_{q}\frac{{z^{\prime }}_{i}}{\|{z^{\prime }}_{i}\|}\frac{k_{b}}{\|k_{b}\|}\right)$$

where the denominator of the Softmax function is the sum of all pre-trained categories. The output of the Softmax function is the probability value of the *b*-th pre-training category, ranging from 0 to 1, representing the coefficient of the weight parameter of the *b*-th pre-training category in the weighted average.

Step 3: Calculate the connection weight parameter value $W^{\prime }$ for the new category of neurons:

(8) $$W^{\prime }=\phi_{\mathrm{avg}}⊙{W^{\prime }}_{avg}+\phi_{att}⊙{W^{\prime }}_{att}$$

where ${W^{\prime }}_{avg}$ is the classifier weight generated based on the average value of features, $\phi_{\mathrm{avg}}$ and $\phi_{att}$ are the trainable parameters, ${W^{\prime }}_{avg}$ is the classification weight generated based on attention mechanism, and ⊙ represents the Hadamard product calculation.

### 2.3. The Prediction Model Based on PCA-Based MTL and AM

When a new operating period of the device begins, the data of new samples that have been standardized can be recorded as a matrix $X_{new}={\left\{x_{1},\cdot \cdot \cdot ,x_{n_{new}}\right\}}^{T}$ of size $n_{new}\times m$, where $n_{new}$ is the number of samples and *m* is the number of features of the monitored device. As previously noted, the direction of the principal component captures the maximum variance of the data, so the projection values in these directions represent the main changes. The projection value of the new sample along the principal component direction is the inner product between the new sample and the principal component, which indicates the relative position of the new sample in this direction. If the projection value of the new sample in the principal component direction is close to the mean of the historical data in the same direction, it indicates that the change in the new sample in this direction is similar to the average change trend of the historical data, which means that the device is operating normally. If the projection is far away from historical data, it indicates that the new sample has undergone significant changes in this direction or is different from the direction of changes in historical data, which means that there may be abnormal operation of the device. The steps for the model to make trend predictions of data from a new operating period are as follows:

Step 1: Standardize the new sample data obtained from a new operating period to obtain $X_{new}$.

Step 2: Calculate the projection value of the new sample $X_{new}$ on the common feature W, and apply the Softmax function to obtain the weight coefficients $\phi =\left[\phi_{1},\phi_{2},\cdot \cdot \cdot ,\phi_{k_{new}}\right]$.

Step 3: Perform dimensionality reduction decomposition on the new sample $X_{new}$ using PCA.

Step 4: Calculate the projection value of the new sample $X_{new}$ on its own load matrix $P_{new}=\left[v_{1},v_{2},\cdot \cdot \cdot ,v_{k_{new}}\right]$ and the number of principal elements $k_{new}$, and apply the Softmax function to obtain the coefficients of its own characteristic direction $\psi =\left[\psi_{1},\psi_{2},\cdot \cdot \cdot ,\psi_{k_{new}}\right]$.

Step 5: Calculate the cosine similarity between each column vector in W and $P_{new}$ to obtain the direction with the highest cosine similarity of the common direction W corresponding to the $v_{1},v_{2},\cdot \cdot \cdot ,v_{k_{new}}$, which is the direction most consistent with the direction in $\beta_{1},\beta_{2},\cdot \cdot \cdot ,\beta_{q}$. Extract $k_{new}$ directions to form a new matrix $W_{1}$ =$\;\left[w_{1},w_{2},\cdot \cdot \cdot ,w_{k_{new}}\right]$, and combine $k_{new}$ coefficients corresponding to $k_{new}$ directions to form a new coefficient matrix $\gamma =\left[\gamma_{1},\gamma_{2},\cdot \cdot \cdot ,\gamma_{k_{new}}\right]$. These directions have similar features or changing trends to the principal component direction being focused in the new sample, which has guiding significance for identifying future data trends and patterns. The common direction $W_{1}$ will have an impact on the feature weight allocation of the prediction model. Thus, it is important to reasonably allocate the weights of the common direction $W_{1}$ and its own feature direction $P_{new}$, which is also the reason for introducing AM.

Step 6: According to the coefficient matrix ψ and γ, calculate the weight coefficient δ1 of the common direction and the weight coefficient δ2 of the feature direction of the new sample and normalize them. Then, use the attention mechanism to adjust weights. Weigh the $W_{1}$ and $P_{new}$ using the formula Z = $\delta 1\cdot W_{1}+\delta 2\cdot P_{new}$ to obtain a matrix Z. Take the first $k_{new}$ columns of the orthogonal matrix of Z as the adjusted eigenvector $P^{\prime }$. The δ1 and δ2 can be calculated as:

(9) $$\delta 1_{i\;}=\frac{\gamma_{i\;}}{\gamma_{i\;}+\psi_{i}},\delta 2_{i\;}=\frac{\psi_{i}}{\gamma_{i\;}+\psi_{i}}$$

Step 7: Project the new sample $X_{new}$ onto the main molecular space to obtain its principal component score $T={P^{\prime }}^{T}X$ and residual amount $E=X-\hat{X}=\left(1-P^{\prime }{P^{\prime }}^{T}\right)X$, where $\hat{X}=P^{\prime }{P^{\prime }}^{T}X$.

### 2.4. Model Monitoring and Evaluation Parameters

After the model training is completed, the operating data of new operating conditions are used as the input for the model to obtain the trend prediction results for the operating data of water pumping station units in the new operating stage. The performance of the model can be monitored and evaluated by Hotelling’s $T^{2}$ and Q statistic during the training and prediction process.

Hotelling’s $T^{2}$ measures the degree of deviation of the sample in multivariate space. It is used to detect abnormal samples in multidimensional data, reflecting the stability of the model. The smaller the value, the more stable the model is. If the $T^{2}$ statistic value of a sample point exceeds the set threshold, the sample point may be considered abnormal. The $T^{2}$ statistic and its threshold $T_{lim}^{2}$ can be calculated by the following formulas:

(10) $$T^{2}=t^{T}\Lambda^{-1}t=x^{T}P^{\prime }L^{-1}{P^{\prime }}^{T}x$$

(11) $$T_{lim}^{2}=\frac{A\left(n_{new}-1\right)}{n-A}F\left(A,n-A,\alpha \right)$$

where *n* is the number of new samples, *A* is the number of principal components extracted by the PCA model, α is the significance level, and $F\left(A,n-A,\alpha \right)$ represents the upper limit of the F distribution with (A,n−A) degrees of freedom, corresponding to the critical value of 100% α.

The Q statistic, also known as squared prediction error (SPE), is a measure of how far the sample deviates from the space of residuals, which is the portion of the space of original variables not explained by the model. The Q statistic is used to monitor the covariance structure of the data. It is calculated through normalization based on the Mahalanobis distance between sample data points and sample means. The statistic Q reflects the predictive accuracy of the model, with smaller values indicating higher accuracy. The statistic Q and its threshold $Q_{lim}$ can be calculated by the following formula:

(12) $$Q=e^{T}e=x^{T}\left(1-P^{\prime }{P^{\prime }}^{T}\right)x$$

(13) $$Q_{lim}=\theta_{1}{\left(\frac{c_{\alpha }\sqrt{2\theta_{2}h_{0}^{2}}}{\theta_{1}}+1+\frac{\theta_{2}h_{0}\left(h_{0}-1\right)}{\theta_{1}^{2}}\right)}^{\frac{1}{h_{0}}},\;h_{0}=1-\frac{2\theta_{1}\theta_{3}}{3\theta_{2}^{2}},\;\theta_{i}=\sum_{j=k_{new}+1}^{n_{new}}\lambda_{j}^{i},i=1,2,3$$

where $c_{\alpha }$ is the standard normal deviation corresponding to upper limit $\left(1-\alpha \right)\times 100\%$. $\lambda_{j}$ is the *i*-th eigenvalue of the covariance matrix of the new sample.

**Figure 2:**
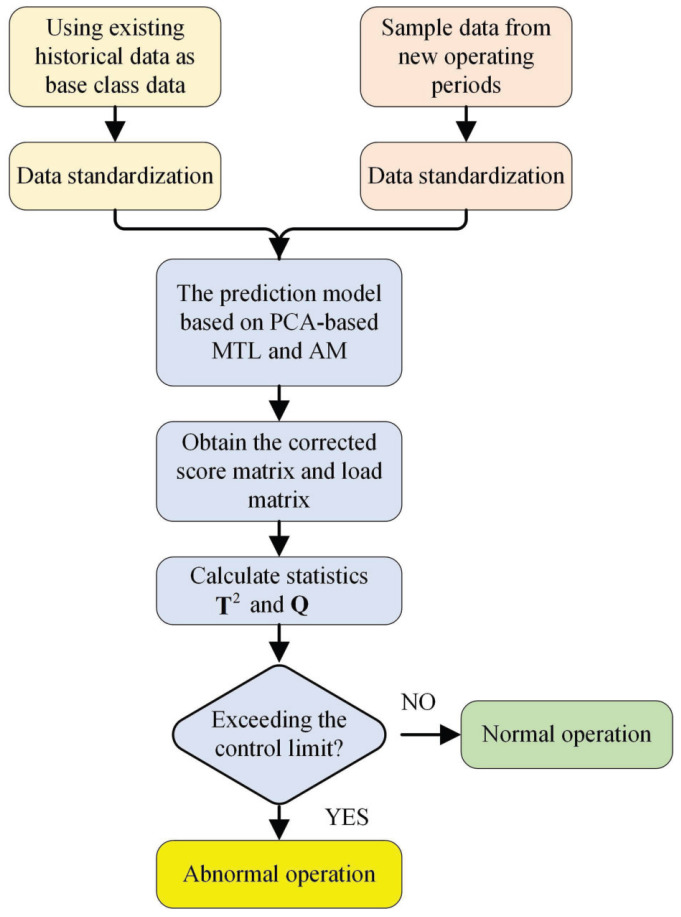
Process of predicting the trend of operating parameters of pump units in pumping stations of the model.

Therefore, the process of predicting the trend of operating parameters for pump units in pumping stations using the PCA-based MTL and AM model is summarized in Figure 2.

## 3. Testing and Results

### 3.1. Data Sources

**Figure 3:**
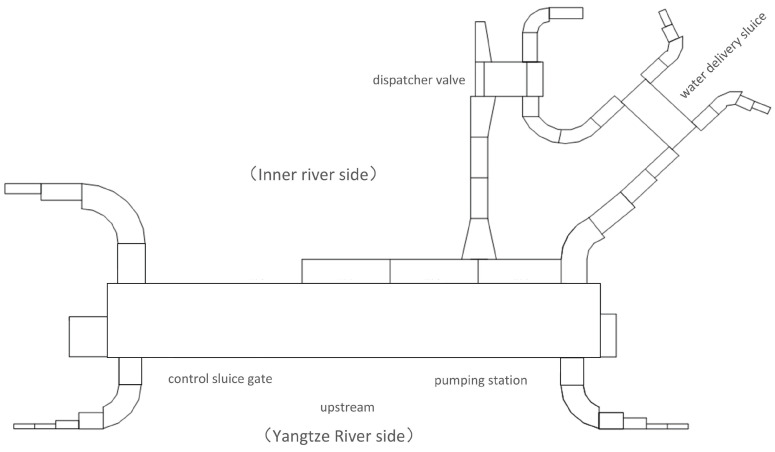
The water level distribution map of the pump station hub.

The PCA-based MTL and attention mechanism (AM) method for predicting the trend of pump station operation parameters, as presented in this paper, primarily focuses on the pump units at a hub along the Yangtze River. Figure 3 shows the water level distribution map of the pump station hub, including the locations of the gate, the pump station, the inner river side, and the Yangtze River side.

**Figure 4:**
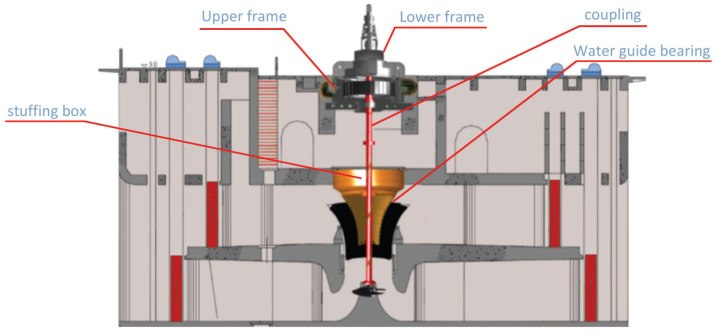
A 3D cross-sectional diagram of the pump unit.

Figure 4 shows a 3D cross-sectional diagram of the pump unit. The pump units in a pumping station are key components of the water resource management system, used to extract, transport, and distribute water resources, ensuring efficient utilization. These pumps can be categorized based on their design and purpose, including as centrifugal pumps, sewage pumps, and self-priming pumps. This paper specifically utilizes a centrifugal pump. Its structure includes a pump casing, impeller, suction inlet, discharge outlet, shaft, and sealing device. The working principle of a centrifugal pump is straightforward and reliable, allowing for efficient liquid transport. This makes centrifugal pumps an ideal choice for various applications, such as water supply, sewage, cooling, chemical processing, and many other fields.

**Figure 5:**
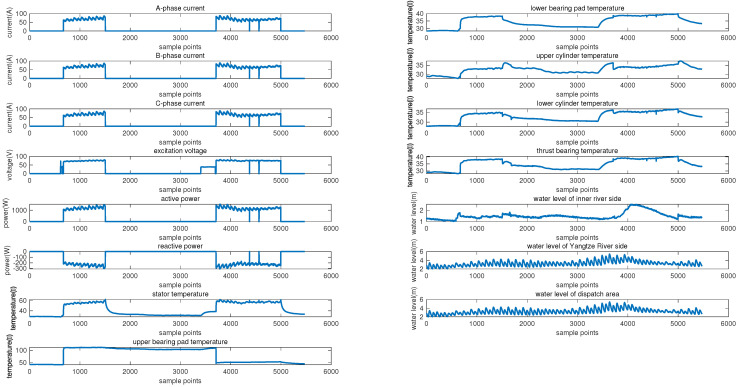
Examples of monitoring data, including: A−phase current, B−phase current, C−phase current, excitation voltage, active power, reactive power, stator temperature, upper conductor temperature, lower conductor temperature, upper cylinder temperature, lower cylinder temperature, thrust tile temperature, inland water level on the inland side, water level on the Yangtze River side, and water level in the dispatch area.

The historical data, comprising 9 sequences and a total of 12,104 sampling points, were collected from 5 units of a pumping station hub along the Yangtze River under normal operating conditions from January to September 2021. These historical data and part of the data from the new operating periods to be predicted were used to extract common features under normal operating conditions as a training set. The data from the new operating periods used for testing were sampled from data of the 1# water pumping, covering the period from 15:50:00 on 5 July 2021 to 08:20:00 on 6 July 2021. A total of fifteen variables were monitored during device operation: the A−phase current, B−phase current, C−phase current, excitation voltage, active power, reactive power, stator temperature, upper bearing pad temperature, lower bearing pad temperature, upper cylinder temperature, lower cylinder temperature, thrust bearing temperature, water level of the inner river side, water level of the Yangtze River side, and water level of the dispatch area. Figure 5 shows examples of all 15 monitoring data. These data are from the 1# pumping unit of the station, and the sampling intervals are from 00:00 on 1 July 2021 to 24:00 on 7 August 2021. Among them, from 15:50 on 5 July to 10:40 on 11 July, and from 18:00 on 26 July to 16:40 on 4 August, the pumping station unit was in operation during these two periods.

In addition, The pump unit operates intermittently and exhibits gradual changes. Each running period can be regarded as each task. Therefore, it is necessary to extract the data of the startup period from the existing data, as shown in Table 1.

Because the training set requires extracting a small number of samples from past stored operational data under different conditions to identify common features, this paper selects nine sequences with relatively larger sample sizes. Specifically, sequences 1–9 from Table 1 are the historical data (also known as the base class), and sequence 10 is selected as the new operating period data to be predicted (also known as the new class).

### 3.2. Model Training

Step 1: Perform the PCA on the base classes of the training set to obtain the number of principal components, the score matrix, and the load matrix, as shown in Table 2, where sequences 1–9 are the historical data and sequence 10 is the partial sample data from the new class.

Step 2: Calculate the cosine similarity between the principal components of the load matrix, set the threshold ς to 0.9, and filter out the principal components of the load matrix with an absolute cosine similarity value greater than ς. There were a total of 11 sets of principal components with consistent directions. By concatenating these 11 sets of principal components with consistent directions and performing singular value decomposition, the common feature matrix **W** of size 15 × 11 could be obtained.

Step 3: By projecting the unit operation data during the new operating period onto the **W** direction, the coefficient matrix ϕ of the unit operation data in the common features directions could be obtained. Similarly, the coefficient matrix ψ of the new data on its own load matrix could also be calculated.

Step 4: The cosine similarity of elements of the common feature matrix and load matrix could be used to filter out the most similar element from the principal components in the load matrix and the direction vectors in the common feature matrix. The cosine similarity of elements of the common feature matrix and load matrix are listed in Table 3, where the cosine similarity marked in orange represents the most similar element direction vector in the common feature matrix with the principal component in the load matrix. Thus, the directions of 5, 1, 3, and 6 from the common feature matrix were selected to compose the new common feature matrix $W_{1}$ = $\left[w_{5},w_{1},w_{3},w_{6}\right]$, and corresponding coefficients were taken from the coefficient matrix ϕ to form the new coefficient matrix $\gamma =\left[\gamma_{5},\gamma_{1},\gamma_{3},\gamma_{6}\right]$.

Step 5: Calculate the weight coefficient δ1 of the common direction and the weight coefficient δ2 to obtain the adjusted eigenvector $P^{\prime }$ as the final feature vector for the PCA mapping, as shown in Table 4.

## 4. Results and Discussion

**Figure 6:**
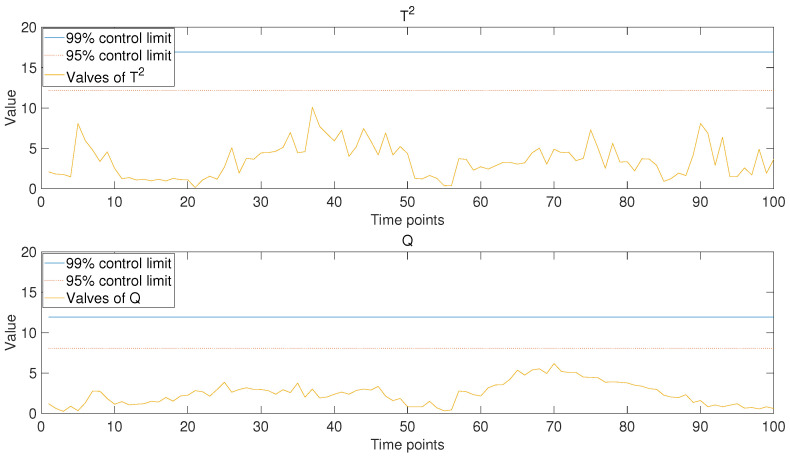
Monitoring results of the model based on PCA-based MTL and AM proposed in this paper. The solid blue line and the dashed red line represent the 99% and 95% control limits, respectively.

**Figure 7:**
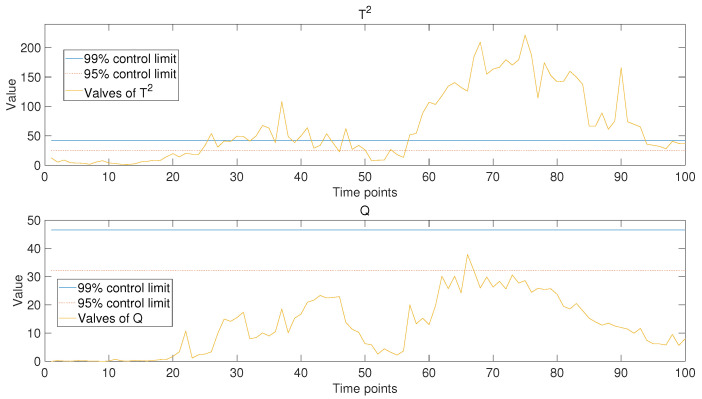
Monitoring results of single-task learning model based on PCA. The solid blue line and the dashed red line represent the 99% and 95% control limits, respectively.

**Figure 8:**
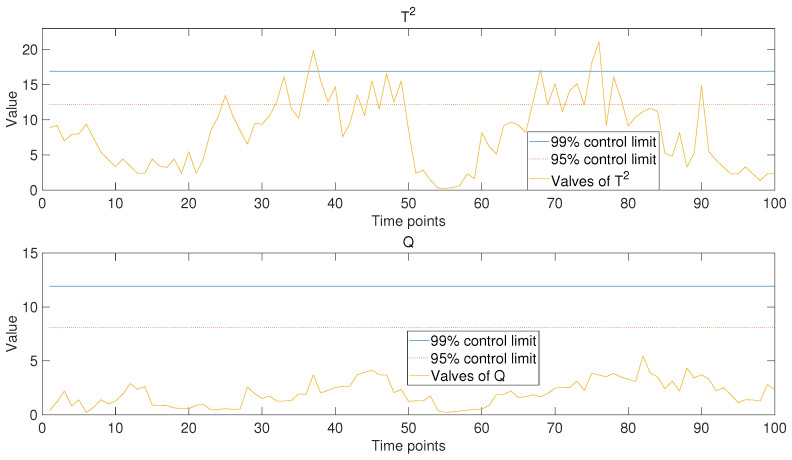
Monitoring results of the PCA-based MTL model without the attention mechanism. The solid blue line and the dashed red line represent the 99% and 95% control limits, respectively.

After the model was trained based on historical data and part of the data from the new operating periods, the operating data of new operating conditions were put into the model to obtain the trend prediction results for the new operating stage. Hotelling’s $T^{2}$ and Q statistics were used to evaluate the model’s performance. Control limits of 99% and 95% were chosen to determine the accuracy of the predictive results. For comparison, in addition to the monitoring results of the model based on PCA-based MTL and AM proposed in this paper (as shown in Figure 6), the analysis results of the other two models were also presented, namely the single-task learning model based on PCA (as shown in Figure 7) and the PCA-based MTL model without the attention mechanism (as shown in Figure 8).

It is not difficult to see from the comparison of the results in the figures that, when the MTL algorithm is not employed (as shown in Figure 7), the values of the monitoring statistics mostly exceed the control limit, indicating a poor stability and prediction accuracy of the model. This result can be easily inferred. Because the single-task learning model based on PCA does not treat each operating stage of historical data as an independent task, it means that it is not able to fully utilize the effective information in historical data. For the PCA-based MTL model without attention mechanism (as shown in Figure 8), part of the statistical values exceed the control limit, indicating that the model cannot fully match the data of the new operating period. This is because common features extracted directly from historical data are used in data mapping, and weight adjustments are not performed based on the characteristics of the data from the new operating period. For the model based on PCA-based MTL and AM proposed in this paper (as shown in Figure 6), the weight of common features had been adjusted by the attention mechanism when conducting data mapping, so the statistics have never exceeded any control limit, which means that the model fits the new operating data very well and predicts the changing trend stably and accurately. By comparing the results, we can draw the following conclusion: the MTL algorithms can fully utilize effective common features in historical data to improve the stability of the model. At the same time, the introduction of the AM could adjust the weights in the data mapping process based on the characteristics of the new operating data to be predicted, thereby improving the prediction accuracy of the model. Additionally, this model can predict the variation trends of multiple parameters in real time, enabling anomaly detection and early warning from multiple perspectives. This significantly reduces issues such as delayed fault identification, missed alarms, and false alarms. In contrast, some existing models or systems tend to focus on the prediction and anomaly detection of single parameters. For example, Hao Zhang et al. achieved fault detection and early warning for units by predicting temperature changes [32], and Jiahao Zhu et al. used VMD and GRU to predict trends in unit vibration signals, enabling the detection and early warning of abnormal conditions [33]. While single-parameter anomaly detection is possible, predicting multiple parameters simultaneously provides a more accurate reflection of the unit’s operating status, leading to the high-precision monitoring of abnormal conditions.

## 5. The Model of Alarm

The statistics of $T^{2}$ and Q could not only be used to evaluate the stability and accuracy of the predictive model but could also serve as judgment thresholds of alarm models to measure whether the devices of the pumping station are operating normally. This paper establishes a multi-level alarm model, which sets different alarm levels based on the threshold of changes in the statistics. Specifically, it is divided into a yellow warning, orange warning, and red warning. The alarm levels and their corresponding thresholds are listed in Table 5.

The purpose of the yellow warning is to remind operation and maintenance personnel to pay attention to the trend of changes in monitoring parameters, which may have potential problems. This warning level does not require immediate emergency measures but requires strengthened monitoring and observation in order to adjust operational strategies in a timely manner. The orange warning indicates that the changes in monitoring parameters have exceeded the normal range and that there is a high possibility of abnormal situations. At this point, operation and maintenance personnel should increase the frequency of inspections and analyze the abnormal causes, and they may take some preventive measures to prevent the problem from further worsening. The red warning indicates that changes in monitoring parameters have significantly exceeded the normal range, potentially leading to serious risks and losses. Operations and maintenance personnel must take immediate emergency measures to stop or adjust the operation to avoid potential accidents and ensure the safety of equipment and personnel. Thus, a multi-level warning system can be implemented to assist operators in responding promptly to different levels of warnings, ensuring the safe and stable operation of the pumping station unit.

## 6. Conclusions

This paper establishes a trend prediction and alarm model for the operating parameters of water pumping stations based on PCA-based multi-task learning and an attention mechanism according to the characteristics of changes in operating parameter data. Compared with traditional single-task PCA models, multi-task learning models can effectively utilize the common features of historical data to predict changes in unit parameters, fully consider the correlation between different tasks, and improve the robustness of prediction models. The introduction of the attention mechanism enables the model to dynamically adjust the mapping weights based on the characteristics of unit operating parameters in new operating periods, thereby further enhancing model stability and prediction accuracy. Based on the model’s prediction results, a multi-level alarm system for monitoring the operation of the unit has been established, which can help operators respond in a timely manner according to different levels of warnings, ensuring the safe and stable operation of the unit, and it has important application value. However, the current PCA-based mapping used to identify common features is linear, while the relationships and interactions among the characteristic parameters of the monitoring data are complex. Therefore, future work could explore nonlinear dimensionality reduction methods to better capture these common features. Additionally, incorporating a broader range of monitoring data could provide a more comprehensive understanding of the operational state of more complex equipment. Finally, exploring multimodal data fusion could further enhance the performance of monitoring systems.

## Figures and Tables

**Table 1:** Existing power-on data statistics table.

Sequences	Unit Number	Power-On Date	Power-On Time	Shut-Down Date	Shut-Down Time	Number of Operating Days	Number of Samples
1	1#	1.29	9:40	2.9	9:30	12	1583
2	1#	7.26	16:00	8.4	16:40	10	1283
3	2#	3.24	15:20	4.2	14:00	10	1291
4	2#	7.26	22:30	8.4	16:50	10	1260
5	3#	1.29	9:40	2.9	9:40	12	1585
6	3#	3.24	15:30	4.2	15:00	10	1294
7	3#	7.26	22:30	8.4	16:40	10	1260
8	6#	7.26	18:10	8.4	16:30	10	1287
9	7#	7.26	22:40	8.4	16:40	10	1261
10	1#	7.5	15:50	7.11	10:40	5	835

**Table 2:** The results of PCA processing on the dataset.

Sequences	Number of Principal Components	Size of the Score Matrix	Size of the Load Matrix
1	4	1584 × 4	15 × 4
2	4	1283 × 4	15 × 4
3	4	1291 × 4	15 × 4
4	4	1260 × 4	15 × 4
5	4	1585 × 4	15 × 4
6	4	1294 × 4	15 × 4
7	3	1260 × 3	15 × 3
8	4	1287 × 4	15 × 4
9	5	1261 × 5	15 × 5
10	4	21 × 4	15 × 4

**Table 3:** The results of the PCA on the base classes of the training set.

		Principal Components in the Load Matrix			
		**Principal Component 1**	**Principal Component 2**	**Principal Component 3**	**Principal Component 4**
**Direction vectors in the** **common feature matrix**	Direction 1	−0.193	**−0.734** ∗	−0.556	−0.095
	Direction 2	0.552	−0.019	0.081	−0.564
	Direction 3	−0.113	−0.367	**0.638** ∗	−0.349
	Direction 4	0.328	−0.654	−0.439	−0.212
	Direction 5	**−0.720** ∗	−0.486	−0.035	−0.282
	Direction 6	0.190	0.007	0.538	**−0.718** ∗
	Direction 7	0.532	−0.165	−0.102	0.074
	Direction 8	0.519	0.230	−0.570	0.407
	Direction 9	0.391	−0.246	0.306	0.266
	Direction 10	0.262	−0.179	0.557	−0.649
	Direction 11	0.227	−0.111	−0.064	0.383

^∗^ The cosine similarity marked in orange represents the most similar element direction vector in the common feature matrix with the principal component in the load matrix.

**Table 4:** Values of weight coefficients δ1 and δ2.

δ1	0.332	0.387	0.009	0.472
** δ2 **	0.668	0.613	0.991	0.528

**Table 5:** The alarm levels and their corresponding thresholds.

Alarm Levels	Alarm Trigger Conditions
The yellow warning	The statistical values are less than the control limit of 95%, allowing for a certain degree of fluctuation.
The orange warning	The statistical values are between the 95% control limit and the 99% control limit.
The red warning	The statistical values are greater than the control limit of 99%.

## Data Availability

The datasets generated during and/or analyzed during the current study are available from the corresponding author on reasonable request.
